# Allosteric activation of RhlB by RNase E induces partial duplex opening in substrate RNA

**DOI:** 10.3389/fmolb.2023.1139919

**Published:** 2023-08-31

**Authors:** Heidi Zetzsche, Laura Raschke, Boris Fürtig

**Affiliations:** Center for Biomolecular Magnetic Resonance (BMRZ), Institute for Organic Chemistry and Chemical Biology, Johann Wolfgang Goethe-Universität, Frankfurt, Germany

**Keywords:** RNA folding, NMR spectroscopy, RNA helicase, real time NMR, RNA dynamics

## Abstract

The *E. coli* DEAD-Box helicase RhlB is responsible for ATP-dependent unwinding of structured mRNA to facilitate RNA degradation by the protein complex degradosome. The allosteric interaction with complex partner RNase E is necessary to stimulate both, RhlB’s ATPase and RNA unwinding activity to levels comparable with other DEAD-Box helicases. However, the structural changes of the helicase RhlB induced by binding of RNase E have not been characterized and how those lead to increased reaction rates has remained unclear. We investigated the origin of this activation for RNA substrates with different topologies. Using NMR spectroscopy and an RNA centered approach, we could show that RNase E binding increases the affinity of RhlB towards a subset of RNA substrates, which leads to increased ATP turnover rates. Most strikingly, our studies revealed that in presence of RNase E (694-790) RhlB induces a conformational change in an RNA duplex with 5’- overhang even in absence of ATP, leading to partial duplex opening. Those results indicate a unique and novel activation mode of RhlB among DEAD-Box helicases, as ATP binding is thought to be an essential prerequisite for RNA unwinding.

## 1 Introduction

The lifetime of a mRNA in prokaryotic cells is very short. Within minutes after transcription, the ribosomes read off and translate the genetic information before the RNA is directly degraded again, only to recycle its nucleotides for the synthesis of the next transcript (Bernstein et al., 2002). This fast-paced cycle allows the cell to quickly respond to environmental and metabolic changes. Responsible for the degradation of mRNA in *E. coli* is an interplay of endoribonuclease RNase E and 3’-5’-exoribonuclease PNPase. The nucleases assemble into a complex called degradosome, with the unstructured C-terminal domain of RNase E functioning as a binding platform for the other complex partners (Py et al., 1994; Bruce et al., 2018). While both RNase E and PNPase play an essential role in degrading the majority of cellular mRNA, they are limited to unstructured RNA substrates and the processivity of PNPase comes to a stop when it encounters sequences that form stem loop structures (McLaren et al., 1991). Therefore, another critical protein in the degradosome complex is the RNA helicase RhlB. RhlB ensures a smooth degradation by unfolding short double-stranded RNA segments in an ATP-dependent reaction. Together with metabolic enzyme enolase the four enzymes form the canonical core composition of the degradosome (Miczak et al., 1996; Py et al., 1996; Bernstein et al., 2004).

RhlB belongs to the DEAD-box helicases, a large family of helicases that can be found in both eu- and prokaryotes and is involved in nearly every aspect of RNA metabolism, from transcription and ribosome biogenesis to translation and RNA decay (Cordin et al., 2006). All DEAD-box helicases share a conserved core structure composed of two linked RecA-like domains (Linder and Jankowsky, 2011). To unwind short RNA duplexes the helicase binds both ATP and the RNA and subsequently induces a strong bent in one of the RNA strands which is incompatible with the duplex helix. This pries several base pairs apart, leading to the dissociation of one strand. The subsequent ATP hydrolysis sets back the protein conformation and both the remaining RNA strand as well as ADP and phosphate are released (Russell et al., 2013).

Several DEAD-Box helicases such as eIF4A or Mss116p have been shown to modulate their activity by complex partners or N- and C-terminal flanking regions (Rudolph and Klostermeier, 2015). In case of RhlB, previous studies demonstrated that the enzyme alone has a barely detectable ATPase activity compared to other DEAD-Box proteins in *E. coli* and that binding the C-terminal domain of RNase E boosts this ATPase and unwinding activity by at least an order of magnitude (Vanzo et al., 1998; Worrall et al., 2008). Based on homology modelling to DEAD-Box helicase Vasa the distance of this interaction side to the catalytic center of RhlB is larger than 20 Å, which raises the question of how the allosteric binding of RNase E translates through the protein to mutually affect the ATP hydrolysis and RNA unwinding.

As the multiple available crystal structures of DEAD-Box helicases reveal, the bound RNA strand is exclusively coordinated via its phosphate backbone and sugar moieties (Sengoku et al., 2006; Del Campo and Lambowitz, 2009; Linder and Jankowsky, 2011). This allows for a discrimination between DNA and RNA molecules but is indicative of an otherwise sequence independent binding. Nonetheless there have been investigations into whether 5’- or 3’-single strand extensions are necessary for efficient helicase activity for different proteins with varying results: while eIF4a and RhlE can unwind both blunt end extended duplexes with a similar performance, a significant drop in ATPase activity was observed for helicases CsdA and SrmB upon elimination of single strand overhangs (Rogers et al., 2001; Bizebard et al., 2004). Whether a DEAD-Box protein requires a certain single strand extension depends apparently predominantly on its function within the cell and must be ascertained for each protein individually. Despite detailed examination of RNA substrate specificity in other *E. coli* DEAD-Box helicases (Bizebard et al., 2004), similar approaches are lacking for RhlB as most ATPase assays utilize yeast bulk RNA as substrate (Vanzo et al., 1998; Worrall et al., 2008). Solely Chandran et al., 2007 examined short duplexes with 3’- or 5’- extension and discovered that RhlBs unwinding rate is significantly higher for a 5’-extended RNA duplex (Chandran et al., 2007).

Here, we set out to probe the mutual influence of different secondary structures and binding to RNase E on the helicase activity of RhlB. To obtain a substrate-centric picture of the unwinding mechanism and its modulation through allosteric binding partners, we investigated the affinity towards RNA, the enzyme kinetics, and the structural changes in the substrate RNA by different NMR spectroscopic methods. NMR spectroscopy provides a unique set of experimental tools to analyse the structure of proteins and nucleic acids with a up to atomic resolution under near physiological conditions. It has therefore been frequently used to investigate contact sites, dynamics and conformational transitions accompanying the formation of RNA-protein or protein-protein complexes of RNA helicases (Oberer et al., 2005; Aumayr et al., 2015; Wiegand et al., 2019).

Our results reveal that the basis of RNase E’s activating effect on RhlB originates in the alteration of RhlBs RNA binding affinity accompanied with changes in the coordination of RNA within the binding pocket. We will also show, how not only these changes in affinity but also RhlB’s RNA substrate preferences translate into differences in ATP hydrolysis rates. This RNA-centric view of RhlB’s enables a better understanding of how structured mRNAs are rapidly degraded within the large degradosome complex centred around RNAse E.

## 2 Material and methods

### 2.1 Preparation of RNA constructs

All unlabelled RNA constructs were purchased from Integrated DNA Technologies, Inc., dissolved in H_2_O and stored at −20°C. The uniformly ^13^C,^15^N-labelled 21 nt RNA single strand (5’ UAG​UAA​CUA​AAA​CAU​UAA​AUU-3’) was prepared as fusion-product with 5’-hammerhead ribozyme (51 nt) and 3’-HDV ribozyme (67 nt) by *in vitro* transcription from a SmaI linearized DNA template (modified pUC57-plasmid) and subsequently cleaved from the ribozymes. Therefore, 100 mM Tris/glutamic acid (pH 8.1), 2 mM spermidine, 40 mM Mg(OAc)2, 15 mM full 13C,15N-labelled rNTPs and 100 ng/µL DNA template were incubated for 30 min at 37°C before 20 mM DTT and 70 µg/mL T7-polymerase (P266L mutant) were added. The incubation was continued for 7.5 h with addition of 1 U/mL yeast inorganic pyrophosphatase (NEB) after 2 h. The RNA product was purified by self-packed anion exchange chromatography using 10 mL DEAE sepharose resin (GE Healthcare) with an elution gradient of 0.6–3 M NaOAc followed by a reversed-phase HPLC using Perfectsil RP18 300A 5 µm 10 × 250 mm column (MZ Analysentechnik) at 60°C, buffer A (50 mM K_2_HPO_4_/KH_2_PO_4_, 2 mM tetrabutylammonium bisulfate, pH 5.9) and buffer B (buffer A+ 60% acetonitrile) in the following sequence of gradients: 0%–37% in 5 min, 37%–40% in 30 min and 40%–100% in 5 min with a flowrate of 5 ml/min. After desalting with Vivaspin™ centrifugal concentrators (Sartorius) and precipitation with 2% (w/v) LiClO_4_ in acetone the RNA pellet was resolved in water and stored at −20°C.

### 2.2 Expression and purification of RhlB and RNase E fragments

Wild type RhlB with C-terminal 6xHis-Tag and TEV cleavage sequence (ENLYFQG) in a pET11A expression vector was purchased by Dharmacon™. Both RNase E (694-790) and RNase E (628-843) were purchased by Dharmacon™ with N-terminal 6xHis-Tag and TEV cleavage sequence in pET21A and pET11A expression vectors, respectively.

Both RhlB and RNase E (628-843) were overexpressed in *E. coli* BL21 (DE3) cells at 37°C using terrific broth (TB) medium and induced at OD600 of 1.5 with 1 mM isopropyl β-d-1-thiogalactopyranoside (IPTG). Expression was continued for 3 h at 21°C before cells were harvested using centrifugation. The cells were resuspended in lysis buffer (500 mM NaCl, 50 mM Tris/HCl, 10 mM 2-mercaptoethanol, 10 mM imidazole, pH 8.3) and EDTA-free protease inhibitor tablet (Roche), lysed using high-pressure homogenization and clarified by centrifugation at 16,000 *g* for 45 min. After addition of 0.03% (w/v) polyethylenimine, the lysate was incubated for 15 min and cleared again using centrifugation. The lysate was loaded onto 5 mL Ni-NTA column (GE Healthcare), washed with lysis buffer first, followed by LiCl buffer (2 M LiCl, 50 mM Tris/HCl, 10 mM 2-mercaptoethanol, 10 mM Imidazole) to remove protein-bound nucleic acids and after a second wash with lysis buffer the sample was eluted with a gradient of 100% Ni-NTA elution buffer over 100 mL (500 mM NaCl, 50 mM Tris/HCl, 10 mM 2-mercaptoethanol, 500 mM imidazole, pH 8.3). The His-Tag was cleaved off through TEV-cleavage during overnight dialysis against 5 L lysis buffer and subsequently removed via reverse Ni-NTA chromatography. The proteins were further purified with a HiLoad 26/60 Superdex 200 column (GE Healthcare) using size exclusion buffer (450 mM KCl, 75 mM Tris/HCl, 5 mM DTT, pH 8.3). The proteins were concentrated to approximately 500 μM, flash-frozen in liquid nitrogen and stored at −80°C. RNase E (694-790) was overexpressed and purified in an analogous manner with the following alterations: 0.4 mM IPTG was used for induction of the pET21A plasmid, lysis and Ni-NTA elution buffer contained 200 mM NaCl, 100 mM KCl, 50 mM Tris/HCl, 5 mM MgCl_2_, 10 mM 2-mercaptoethanol at a pH of 8.0 and 5 mM or 500 mM imidazole, respectively, and a HiLoad 26/60 Superdex 75 column (GE Healthcare) was used for size exclusion chromatography.

For both NMR experiments and assays, suitable amounts of proteins were thawed and rebuffered into NMR buffer (150 mM KCl, 25 mM Tris, 5 mM DTT, 4.5 mM MgCl_2_, pH 8.3) using Vivaspin™ centrifugal concentrators.

### 2.3 Electrophoretic mobility shift assay

Binding reactions were carried out in 150 mM NaCl, 25 mM Tris, 1.5 mM MgCl_2_, 5 mM DTT, 10% glycerol at pH 8.3. 20 μL reaction were prepared with 1.5 µM RNA and increasing amounts of RhlB and incubated for 15 min at 4°C. 5 μL of each sample was loaded onto a layered 6% + 10% native acrylamide gel (29:1 ratio acrylamide to bisacrylamide) running at ∼70V in TA buffer (40 mM Tris, 0.1% (v/v) acetic acid, pH 8.0) for 5 h at 4°C. The gel was stained with GelRed staining solution (0.0001% (v/v) GelRed^®^ Nucleid Acid Gel stain in water) for 15min and RNA bands visualized via UV-transillumination using a Gel iX20 Imager (Intas Science Imaging). For protein visualization, gels were afterwards stained with Coomassie staining solution (10% (v/v) ethanol, 5% (v/v) acetic acid, 0.0025% (w/v) Coomassie brilliant blue G250, 0.0025% (w/v) Coomassie brilliant blue R250) and the gel digitalized by Gel iX20 Imager. The fraction of bound RNA was calculated by quantifying the intensity of the free RNA band relative to the intensity of the “0” lane containing only the RNA using ImageJ (after subtracting the background). The fraction of bound RNA was plotted against protein concentration and the data were fitted for a simple single binding site function y=(B*x)/(K_*D*_ *+x)*, where B is the upper plateau of the binding curve.

### 2.4 ATPase assay

ATPase activity of RNA helicase RhlB was determined spectrophotometrically using the Molecular Probes EnzChek Phosphate Assay kit (Invitrogen), which is based on a method originally described by Webb (Webb, 1992). Here, the original kit layout was modified through downscaling of the final reaction volume to 200 µL and transfer of the cuvette-based assay layout to 96-well microplates. Assays were performed in 111 mM KCl, 68.5 mM Tris, 1 mM MgCl_2_ and 0.1 mM sodium azide at pH 8.1 with 6 µM of the corresponding RNA substrate and 2.4 µM RhlB (and RNase E fragment). Following preincubation of 160 µL of the reaction components for 10 min at 22°C, the reaction was started through addition of 40 µL ATP to a final concentration of 0.2 mM. Reactions were monitored at 360 nm for 300 s with an Infinite^®^ 200 PRO microplate reader (Tecan), measuring triplicates of the desired sample composition. The modified reaction volume and vessel required the conversion of the absorption raw data with the correct optical path length according to Lambert-Beer. The initial reaction rates were calculated via linear regression of the rate curve from 120 to 220 s and was converted into “mol phosphate min-1 mol helicase-1” with a phosphate standard curve.

### 2.5 NMR spectroscopy

Unless stated otherwise, all NMR samples were prepared in NMR buffer (150 mM KCl, 25 mM Tris/HCl, 5 mM DTT, 4.5 mM MgCl_2_, pH 8.3) with 6%–10% D_2_O and 100 µM sodium trimethylsilylpropanesulfonate (DSS) as well as 0.3–1 mM phosphocreatine (PCr) were used as chemical shift standards for ^1^H and ^31^P experiments. NMR experiments were performed at 288 K either on a Bruker AV 600 equipped with a TCI-HCP probe, a Bruker AV II 600 equipped with a TCI-HCN probe, a Bruker AV III 600 equipped with a TCI-HCN probe, a Bruker AV III 700 equipped with a QCI-HCNP probe or on a Bruker AV 800 equipped with either a TXO-HCN or TCI-HCN probe (Rheinstetten, Germany) and processed with Topspin 4.0.8 (Bruker, Rheinstetten, Germany).

### 2.6 ^31^P real-time NMR mixing experiments

For NMR real-time mixing experiments a 300 µL volume of 100 µM unlabelled 5’-OV RNA construct, 400 µM RhlB (+/−400 µM RNase E), 1 mM phosphocreatine, 100 µM DSS and 6% D_2_O were prepared in NMR buffer in a 5 mm Shigemi NMR tube and inserted with a glass capillary containing 40 µL of injection solution (25.5 mM ATP in NMR buffer with 6% D_2_O). The rapid-mixing setup, as illustrated in Figure 3, was adapted from Mok et al. (Mok et al., 2003). 32 k consecutive scans of ^31^P 1D spectra were recorded as a pseudo 2D at 288 K, with the ATP injection being triggered after 128 scans. The amount of ATP was determined from peak integrals of ATPα and the resulting curves fitted with double exponential curve fit.

### 2.7 1D and 2D NMR titration experiments and K_D_ determination

For ^1^H and ^13^C HSQC titration experiments 180 µL of 100 µM unlabelled or double labelled RNA substrate (5’-UAG​UAA​CUA​AAA​CAU​UAA​AUU-3’ fully 13C, 15N labelled), 100 µM DSS and 6%–10% D_2_O were prepared in NMR buffer in 3 mm NMR tubes and unlabelled protein was added in steps of 0.25, 0.5, 1, 2 and 4 equivalents over RNA. The total protein stock concentration varied between 0.75 and 2 mM depending on intrinsic stability of RhlB and RNase E fragments and dilution effects in the spectra were corrected with DSS reference signal.

For K_D_ determination of ^1^H titration the normalized reciprocal peak intensities of non-overlapping imino proton resonances were plotted against protein concentration. The maximum decrease was determined in the experiment with the highest protein concentration. The resulting curves were fitted globally based on a single-site ligand binding equilibrium (Fielding, 2007; Williamson, 2013).

$${∆}_{obs}={∆}_{\max}\frac{\left(K_{D}+{\left[P\right]}_{0}+{\left[R\right]}_{0}\right)-\sqrt{{\left(K_{D}+{\left[P\right]}_{0}+{\left[R\right]}_{0}\right)}^{2}-\left(4{\left[P\right]}_{0}{\left[R\right]}_{0}\right)}}{2{\left[R\right]}_{0}}$$

With K_D_ being the apparent K_D_ value, [P]_0_ and [R]_0_ the protein and RNA concentrations respectively.

## 3 Results

### 3.1 Influence of substrate RNA topology and RNase E fragments on ATPase activity

**FIGURE 1:**
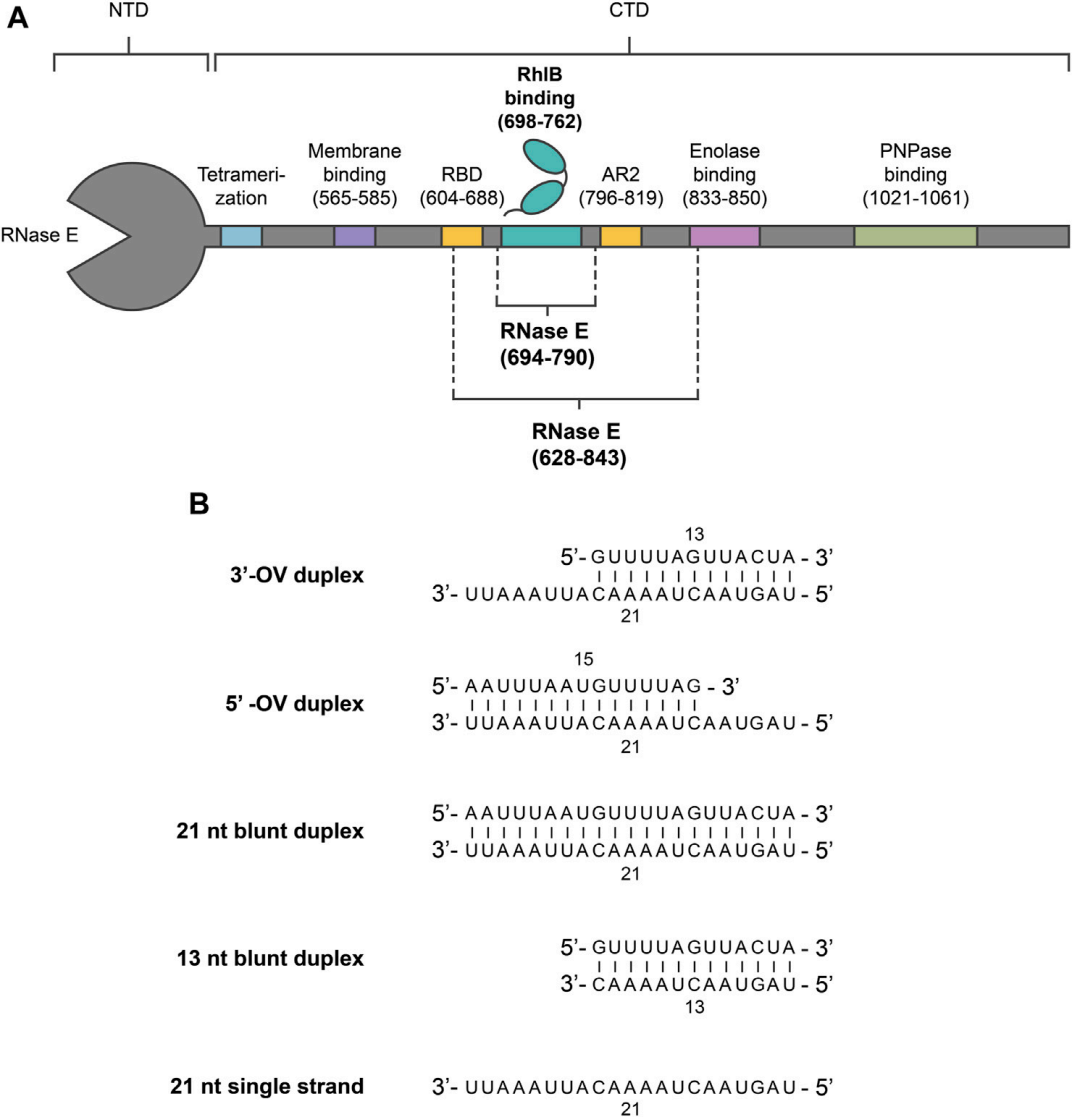
Overview of RNase E and RNA constructs used in this study. **(A)** Oligonucleotide sequences of the used RNA duplex and single strand constructs. For a single stranded substrate, a 21 nt long oligonucleotide strand was utilized. Pairing the 21 nt strand with complementary strands of different length resulted in the 3’-OV, 5’-OV and 21 nt blunt end constructs. The 13 nt blunt end construct was designed by truncating the overhang of the 3’-OV construct. **(B)** Schematic representation of *E. coli* RNase E and RhlB. RNase E’s interaction sites with protein complex partners (RhlB, Enolase and PNPase), RNA (RBD, AR2), membrane and other degradosome monomers (tetramerization site) within the carboxy-terminal domain (CTD) are indicated. The RNase E fragments used are RNase E (694-790) and RNase E (628-843), which have been previously studied by other groups (13) (18). RNase E (694-790) encompasses the binding site for RhlB (698–762), whereas RNase E (628-843) also includes RNA binding sites AR2 and parts of RBD.

Understanding the molecular mechanism of RhlB requires insight into the influences of its substrate RNA structure and the allosteric regulatory effects of its interaction partner RNase E on its catalytic activity. As shown by Worrall et al., ATP hydrolysis assay delivers a direct readout of the catalytic performance of the helicase (Worrall et al., 2008). Here, we study a variety of different RNA substrates with a specific and defined structure, thereby reaching a topological resolution that is inaccessible by older studies that relied on testing bulk RNA from *S. cerevisiae*. The individual RNA constructs (Figure 1A) represent RNA substrates that differ in features like length (13–21 nt) or single strand extensions and include an RNA single strand. We probe those different RNA substrates for RhlB since distinct preferences for particular RNA features are often closely connected to the cellular functions of DEAD box helicases (Tsu et al., 2001; Bizebard et al., 2004). To be compatible with subsequent NMR spectroscopic analysis, the RNA constructs were chosen to be smaller than 50 nucleotides to facilitate sequence assignment and to be composed of two individual strands to allow for strand-selective isotope labelling. Furthermore, the used sequences were derived from an RNA that showed significant catalytic activity in *vitro* studies of the *E. coli* DEAD-box helicase CsdA compared to similar structured sequences with a higher GC-content (Stampfl et al., 2013).

**FIGURE 2:**
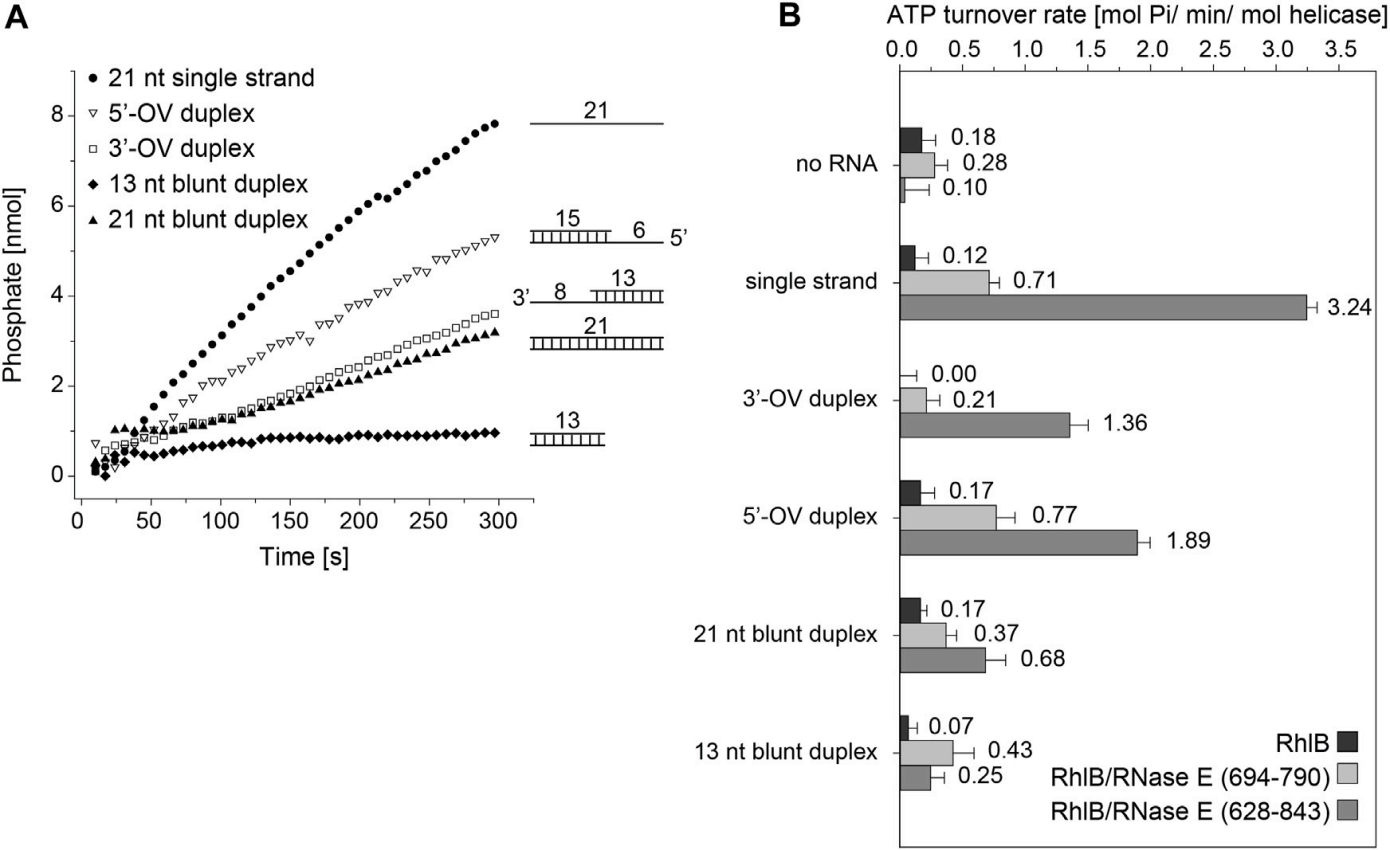
ATPase activity of RhlB with varying RNA substrates and RNase fragments. **(A)** Representative activity profile of RhlB in complex with RNase E (628-843) and single stranded (●), 5’ overhang (△), 3’ overhang (□) 13 nt (♦) or 21 nt blunt end (▲) RNA substrates. **(B)** Graphical representation of ATP hydrolysis rates of RhlB in presence and absence of RNase E (694-790) or (628–843) as well as different RNA substrates.

The ATP turnover rates measured for RhlB with any of the RNA substrates shows no significant rate increase over the control measurement without RNA and only minute differences between the individual substrates (Figure 2). This minimal ATP hydrolysis rates of RhlB are in agreement with Worrall’s findings who in the presence and absence of RNA also detected activity rates below 0.5 mol Pi min^-1^ mol helicase^−1^, ranging borderline to the assays sensitivity (Worrall et al., 2008).

We also probed RhlB’s ATPase activity in complex with the minimal binding fragment of RNase E, that has been shown to bind and stimulate RhlB’s ATP turnover rate (RNase E (694-790); see Figure 1B) (Worrall et al., 2008). Hetero-complex formation in 1:1 stoichiometry between RhlB and RNase E (694-790) was confirmed through analytical size exclusion chromatography (Supplementary Figure S1). While a clear increase in ATPase activity are observed for all RNA substrates, there are notable differences in the degree of activation. The measurements with RNA featuring a 3’-single stranded overhang (3’-OV) exhibit only minor rate increase whereas the experiments with both the single stranded RNA as well as the RNA construct with a 5’-single stranded overhang (5’-OV) show considerable activity enhancement. Notably, those are the only two constructs that feature a single stranded 5’-end. Both blunt end RNA duplexes perform similar regarding turnover rate and display an activation slightly lower than the single strand.

With the addition of RNase E (628-843), a larger fragment encompassing the two RNA binding sites RBD and AR2 (See Figure 1B), an even stronger activating effect could be detected on reactions with both single strand overhang RNA substrates as well as the single stranded RNA (while both blunt end constructs exhibit only minimal or no rate increase). Interestingly, the 13 nt blunt RNA construct even shows a reduction in turnover rate in the presence of RNase E (628-843), whereas 3’-OV RNA, the same construct only extended by a single strand overhang shows a dramatic rate increase. The sheer length of RNA strand being the defining factor for the turnover rate can be discarded as measurements with the 21 nt blunt end construct again exhibit significantly less rate increase than the 3’-OV RNA construct.

In total, RNase E (694-790) and (628–843) increase the ATP turnover rate by a factor of up to 6 and 27, respectively. From these experiments, it is apparent that the ATP hydrolysis reaction is driven towards the product side of the reaction either by single stranded RNAs or 5’-single stranded RNA fragments. This finding is corroborated by the finding that the RNase E (628-843) which contains binding sites for RNAs has an enhancing effect, that exceeds the pure allosteric enhancement that is exhibited by RNase E (694-790).

### 3.2 RNase E dependent ATPase activation of RhlB under NMR conditions

**FIGURE 3:**
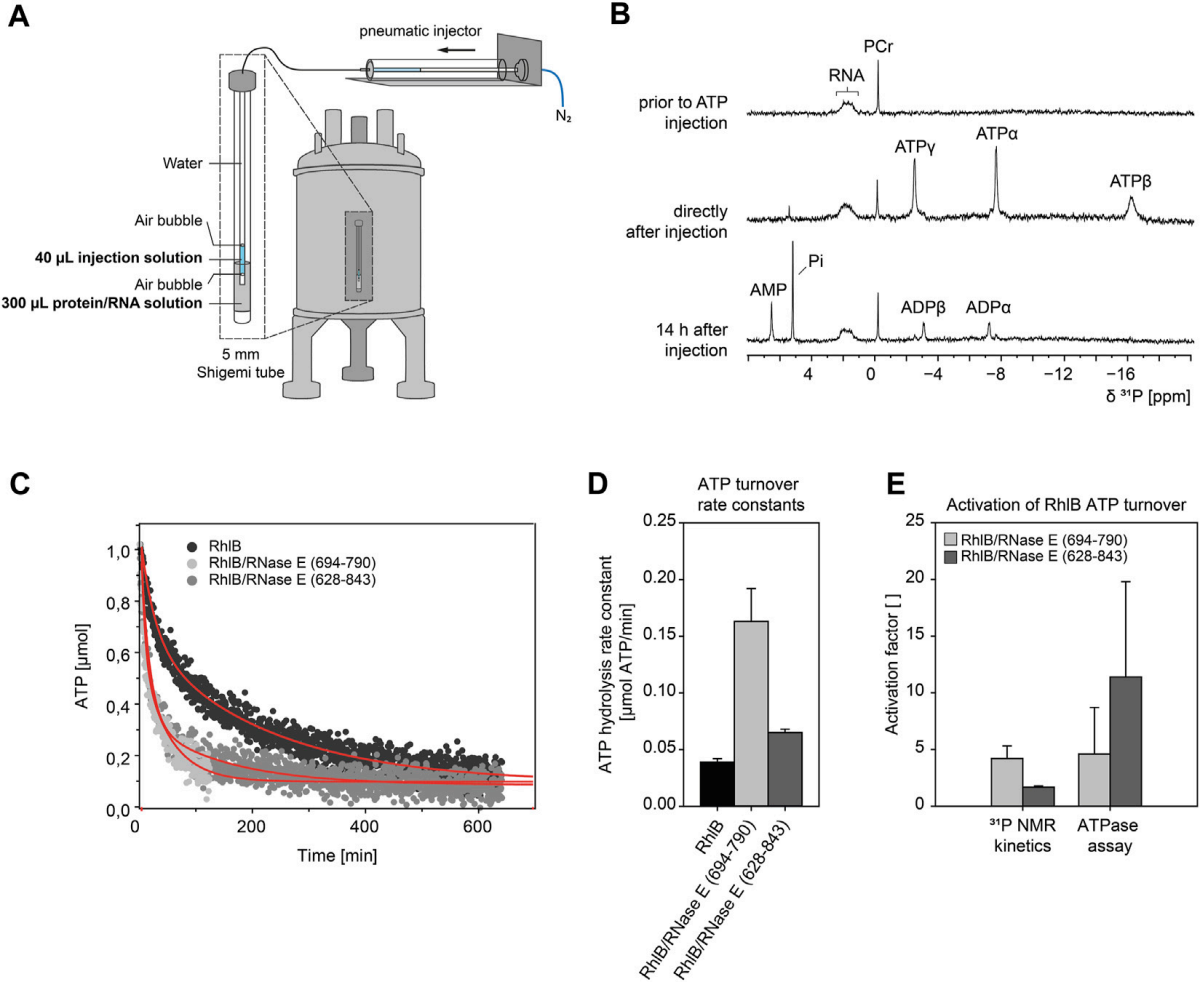
ATP hydrolysis kinetics measured with ^31^P NMR real-time mixing setup. **(A)** Schematic of a real-time NMR mixing setup including NMR tube with injection insert. Injection of ATP solution is triggered by pneumatic piston in direct response to electronic signal of pulse sequence command. The 5 mm NMR shigemi tube containing a preequilibrated protein/RNA mix (100 µM 5’-OV RNA duplex, 400 µM RhlB or RhlB/RNase E complex (1:1), 100 µM DSS, 6% D_2_O, 150 mM KCl, 25 mM Tris/HCl, 5 mM DTT, 4.5 mM MgCl_2_ at pH 8.3) was mixed with 40 µL of injection solution (25.5 mM ATP, 100 µM DSS, 6% D_2_O, 150 mM KCl, 25 mM Tris/HCl, 5 mM DTT, 4.5 mM MgCl_2_ at pH 8.3) to result in a final ATP concentration of 3 mM. **(B)** 1D^31^P NMR spectra of the protein/RNA reaction mix prior to ATP injection, directly after injection (4.2 min) and 14 h after injection. The appearance of ATP resonance signals confirms the successful injection process. Spectra were recorded with 128 scans and referenced to phosphocreatine (PCr). Peak assignments: Pi, orthophosphate; AMP, α-phosphate group of adenosine monophosphate; ADPα/ADPβ, α- or β-phosphate groups of adenosine diphosphate; ATPα/ATPβ/ATPγ, α-, β- or γ-phosphate groups of adenosine triphosphate. **(C)** Kinetic NMR data of RhlB induced ATP hydrolysis in ^31^P real-time NMR experiment. Shown total amount of ATP over time for reactions with RhlB alone and in complex with RNase E (694-790) or RNase E (628-843). The amount of ATP was determined from ^31^P peak integral of ATPα and curves were fitted with double exponential curve fit. Experiments were recorded for 12.8 h as pseudo-2D with 32k scans and ATP injection after 128 scans. The measurement with RNase E (694-790) was discontinued at 124 min and fitted for that duration. **(D)** Bar diagram of ATP turnover rate constants k_1_ extracted from double exponential fits. **(E)** Relative NMR rate constants and ATP hydrolysis assay rates expressed as activation factor with respect to RhlB only measurement.

NMR spectroscopic experiments are very different from UV/Vis based assays (Bains et al., 2019). Sample volumes and concentrations increase by at least an order of magnitude. The experimental setup requires changes in the ratio of interacting biomolecules as the solubility limit can be easily reached. In order to verify that our subsequent NMR measurements correctly describe the molecular effects observed in the ATP hydrolysis assay we performed real-time NMR mixing experiments with the detection of the ATP turnover in consecutive ^31^P 1D NMR spectra (Figures 3A,B). Right after the reaction is initiated by injection of ATP into a solution of pre-equilibrated RhlB/5’-OV-duplex RNA complex, the ATP signals that were tracked over up to 12.8 h showed a rapid decrease in intensity, as depicted in Figure 3C for ATPα. Simultaneous increase in resonances specific for ADP and Pi confirm that ATP is converted into ADP and orthophosphate. Control experiments with ATP in buffer without RhlB show that ATP is stable at room temperature for at least 24 h and is not prone to spontaneous degradation under measurement conditions (see Supplementary Figure S2). From that we can infer that the ATP hydrolysis observed in the mixing experiments is in fact caused by enzymatic turnover by RhlB. ADP itself is significantly less stable under this buffer conditions and therefore hydrolyses further into AMP and orthophosphate, which can both be detected after 14 h in solution as well (see Figure 3B). The experiments with a complex of RhlB and either RNase E (694-790) or RNase E (628-843) both show an accelerated ATP turnover in comparison to RhlB alone (Figure 3D) and are therefore indicating that the activating effect of RNase E can in fact be reproduced under NMR conditions. In the ^31^P real-time NMR experiments and in the ATPase assay the stimulating effect of RNase E (694-790) is comparable with factors between four and five. However, for the RhlB complex with the larger fragment RNase E (628-843) is rather minute (facor of ≈2) in the ^31^P real time NMR experiment compared to the phosphate assay (factor of ≈10) (Figure 3E). We attribute this deviation to the altered protein:RNA ratio from 4:10 in the phosphate assay to 4:1 in the NMR experiments. Changing the ratio was necessary to ensure complete binding of RNA to RhlB since the RNA concentrations for this experiment are close to RhlB’s K_D_ (see following section). Further, RNAse E (628-843) contains two RNA binding sites flanking its RhlB binding site. As described in the following section, the affinity for RNA binding in RNAse E (628-843) is given by a K_D_ of 162 ± 30 μM, so that under the given excess of proteins over RNA these RNA binding regions of RNase E (628-843) compete with RhlB for the RNA substrate. Therefore, we assume that this will lead to an decreased amount of RNA available for binding the helicase in the active site.

### 3.3 RNase E selectively increases RNA binding affinity of RhlB

**FIGURE 4:**
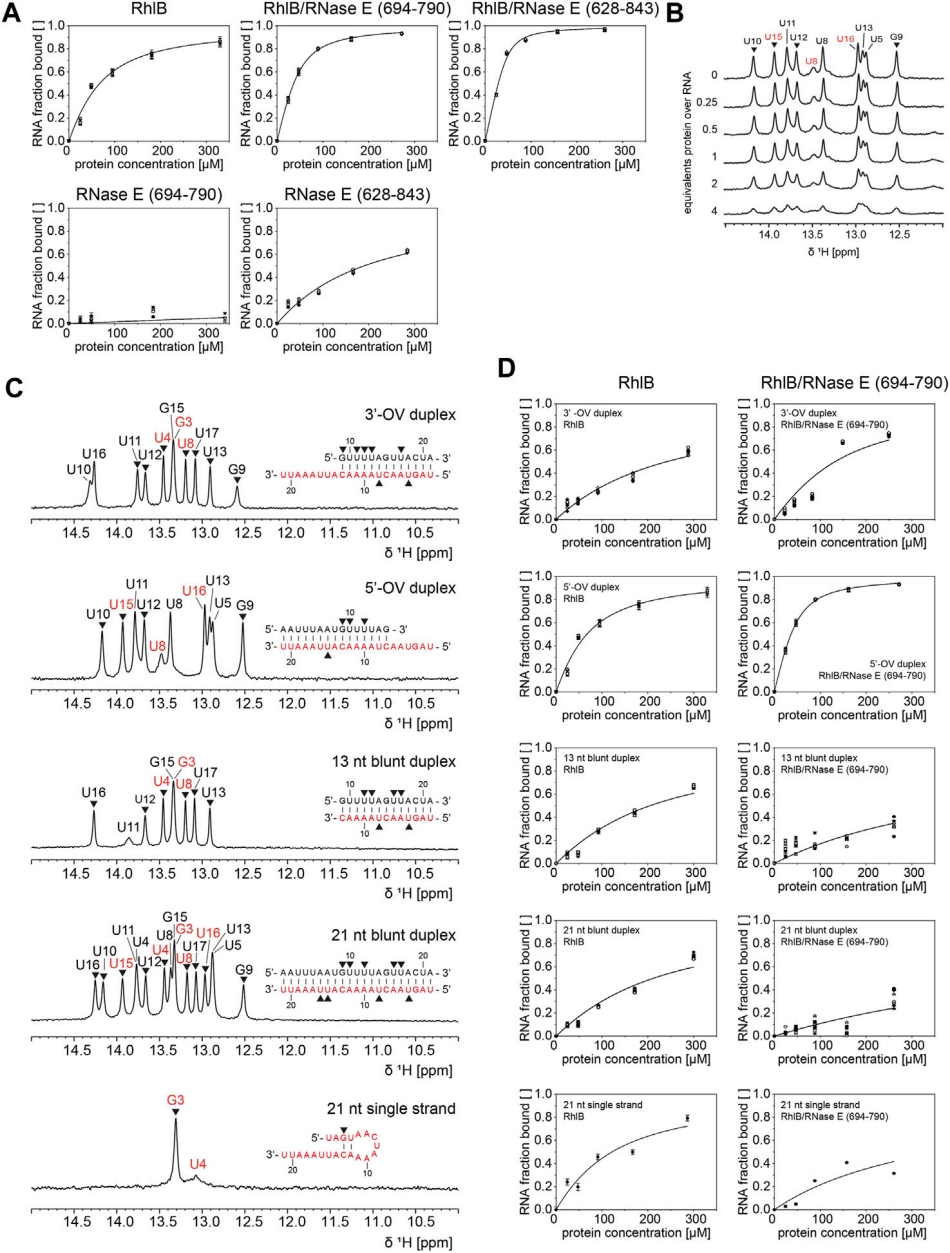
^1^H NMR analysis of RhlB’s binding affinity towards different RNA substrates in presence and absence of RNase E (694-790) or (628–843). **(A)**. RNA binding curves of 5’-OV RNA substrate for titrations with RhlB alone, with RhlB in complex with RNase E (694-790) or RNase E (628-843), and with RNase E (694-790) or RNase E (628-843) alone. **(B)** Exemplary imino proton region of the 1D ^1^H NMR spectra of the 5’-OV RNA substrate with stepwise titration of up to 4 equivalents RhlB. **(C)** Assigned imino proton region ^1^H NMR spectra for all RNA substrates including oligonucleotide sequences. It is noted that the 21 nt single strand forms two weak base pairs under NMR conditions. Resonances assigned to bottom or top strand are colour coded in red and black, respectively, and only peak intensities of non-overlapping resonances (annotated with ▼) were used for K_D_ calculations. **(D)** RNA binding curves of all RNA substrates for titrations with RhlB and RhlB/RNase E (694-790). Binding affinities were determined by plotting normalized reciprocal peak intensity against protein concentration and fitting globally with ligand binding function (Fielding, 2007).

To understand where both the RNA-substrate-dependent differences in ATP turnover as well as the RNase E activation have their origin, we investigated the binding of RNA to the helicase by performing 1D 1H NMR titration experiments. Monitoring the intensity and the overall changes of RNA imino proton resonances upon addition of RhlB does not only allow for determination of an apparent K_D_ but also reveals possible structural rearrangements and changes in base pairing within the RNA substrate during binding. For all of the five different RNAs the imino protons could unambiguously be assigned and therefore their conformation and topological configuration could be determined (Figure 4C).

As exemplified in Figure 4B, the peak intensities of all non-overlapping imino proton resonances were tracked during titration of up to 4 equivalents of protein over RNA and the normalized reciprocal peak intensity was plotted against the protein concentration to extract apparent K_D_ values (Williamson, 2013).

For every titration we could observe a homogenous intensity decrease accompanied by peak broadening, which is indicative of binding to the large protein as this affects the tumbling speed of the RNA and therefore the linewidth of the peak. The reduction of intensity of the imino proton resonances could of course also be explained by an increased population of non-base paired conformations such as a partial or fully single stranded conformation. However, the formation of such a conformation is typically not observed as a uniform intensity decrease for all the imino resonances. Furthermore, the induction of a second conformation with open base pairs has to be detectable through significant chemical shift changes for the resonances of the non-exchanging atoms in the RNA. As this is only detectable for the RhlB/RNAse E complexes but nor for RhlB alone, it is fair to assume that the reduction of intensity of imino proton resonances stems dominantly from the binding interaction rather from opening of the base pairs. Furthermore, the surprising lack of chemical shift perturbation for the imino proton signals might originate from the proposed binding mode of the helicase, that rather influences signals stemming from the sugar phosphate backbone than those of the imino-protons involved in central helical hydrogen bonds. The stoichiometry and the exact nature of the formed RNA-helicase complexes will also influence the analysis of the binding affinity. In order to validate our first approximation that the reduction of signals can be related to the binding of RNA by RhlB, we compared the binding curves derived from the NMR experiments with those obtained in an electrophoretic mobility shift assays for a subset of the constructs (see Supplementary Figure S5). The comparison shows that within experimental error both methods yield the same apparent K_D_ values and show that the NMR based method correctly reports on the formation of the RNA-helicase complex. However, with both methods the detection of the exact stoichiometry of the complex is hard to assess. Nevertheless, given the size of the used RNAs the formation of higher order complexes is rather unlikely, as others have reported complex ratios of. 1:1 for analogue DEAD-Box helicases with similar sized RNAs and none of the highly conserved DEAD-Box helicases is known to multimerize (Bizebard et al., 2004; Bono et al., 2006; Sengoku et al., 2006; Collins et al., 2009).

The 5’OV RNA was titrated against RhlB and its different RNAse E complexes, furthermore also the two RNAse E fragments of different length were separately tested for their affinity to RNA (Figure 4A). All the other RNAs under study were titrated with RhlB and the RhlB/RNAse E (694-790) complex, as this is the most active complex in the NMR-based ATPase assays (Figure 4D).

As summarized in Table 1, for RhlB we could measure a low micromolar K_D_ of 46.6 µM for the 5’-OV RNA construct, which is three times weaker than the K_D_ measured for the RhlB/RNase E (694-790) complex. Since the control experiment with RNase E (694-790) alone provides evidence that this unstructured protein fragment does not bind RNA, we conclude that the interaction of RNase E (694-790) with RhlB allosterically affects the affinity of the helicase for RNA. Analogous experiments with RNase E (628-843) show that the RNA binding regions RBD and AR2 bind the 5’-OV RNA with a K_D_ of 162.7 µM. Due to the unstructured nature of those binding sites as well as the high density of positively charged amino acids this is most likely a non-specific electrostatic interaction with the negatively charged RNA backbone (Callaghan et al., 2004; Bruce et al., 2018). The complex of both RhlB and RNase E (628-843) have a combined apparent affinity of 5.1 µM. Taken together, this data illustrate how the two individual effects of allosteric activation and RNA binding regions contribute to RhlB’s interaction with RNA.

**TABLE 1:** Binding affinity of RhlB for five different RNA substrates in the presence and absence of RNase E (694-790) and RNase E (628-843).

	Apparent K_D_ [µM]^a^				
RNA substrate	RhlB	RhlB/RNase E (694-790)	RhlB/RNase E (628-843)	RNase E (694-790)	RNase E (628-843)
5’-OV duplex	46.6 ± 6.8	14.5 ± 0.7	5.1 ± 0.8	n.b.^b^	162.7 ± 30.9
3’-OV duplex	216.3 ± 33.7	82.3 ± 27.3			
Blunt duplex 13 nt	154.9 ± 33.7	487.5 ± 40.5			
Blunt duplex 21 nt	161.7 ± 30.3	n.d. **			
21 nt single strand	94.9 ± 59.2	264.5 ± 1.0			

^a^ The errors are the mean ± S.D.

^b^ no binding observed.

We were also able to detect significant differences in RhlB’s affinity towards the investigated RNA substrates. Most strikingly, RhlB binds the 3’-OV RNA construct with a K_D_ of 216.3 µM, which is 4.5 times weaker than the affinity towards the same sized 5’-OV RNA construct and also significantly weaker than the K_D_ of 94.9 µM for the single stranded RNA substrate. RhlB’s affinity towards both blunt end constructs appear to be between that of the single strand and the 3’-OV RNA with the difference in duplex length not having a significant effect on the overall affinity. Those results strongly indicate a substrate preference of RhlB towards constructs with a single stranded 5’-end. The differential affinity of RhlB towards topological different RNAs might be the origin for the observed differences in the unwinding rates (Chandran et al., 2007).

Interestingly, the addition of RNase E (694-790) to the helicase does not increase the affinity for all RNA substrates in a similar manner. In fact, the affinity for both blunt end constructs as well as the single stranded construct decreased upon titration of the RhlB/RNase E (694-790) complex, in case of the 21 nt blunt end duplex even beyond detectability by NMR. Both 3’- and 5’-OV RNA on the other hand show a distinct increase in affinity, suggesting that interaction partner RNase E (694-790) narrows RhlB’s RNA substrate preferences.

Blunt ended doubled stranded RNAs are accepted as substrate, but the proper native substrate are double stranded regions with 5’-single stranded overhangs as RNAs with this topology not only exhibit the highest ATP turnover rates but also the highest affinity.

### 3.4 Induction of a partially single stranded conformation in the substrate RNA

Besides monitoring the mere binding to the proteins, NMR is also capable to detect the induction of new conformational states within the RNA that is being bound to the helicase and its RNAse E complexes. NMR is thereby capable of discriminating the base pairing state at nucleotide resolution and even if the base pairing states are only populated transiently.

Upon binding of RhlB, no site-specific effect affecting only a subset of the imino-proton resonances could be observed. This can indicate that either the imino-protons are too distant from RhlB’s actual binding site on the sugar-phosphate backbone or that structural changes like base pair opening are induced at the fraying blunt end of the RNA helix, that does not exhibit detectable imino-proton resonances.

We therefore chose to perform 2D NMR titration experiments using ^13^C labelled RNA substrates (21 nt strand being fully ^13^C labelled) to gain structural insights into the effects of RNase E (694-790) on the RNA during binding. ^13^C-HSQC measurements give us a variety of possible RNA reporter signals in close proximity to the proposed interaction points whose peak intensity is unaffected of pH dependent solvent exchange. Furthermore, the chemical shifts of those resonances are sensitive to their chemical surrounding and the RNA’s conformation. We concentrated our evaluation on nucleobase resonances C6H6, C8H8 and C2H2, as the spectral overlap and resolution of other signals impede a complete tracking of the resonances.

**FIGURE 5:**
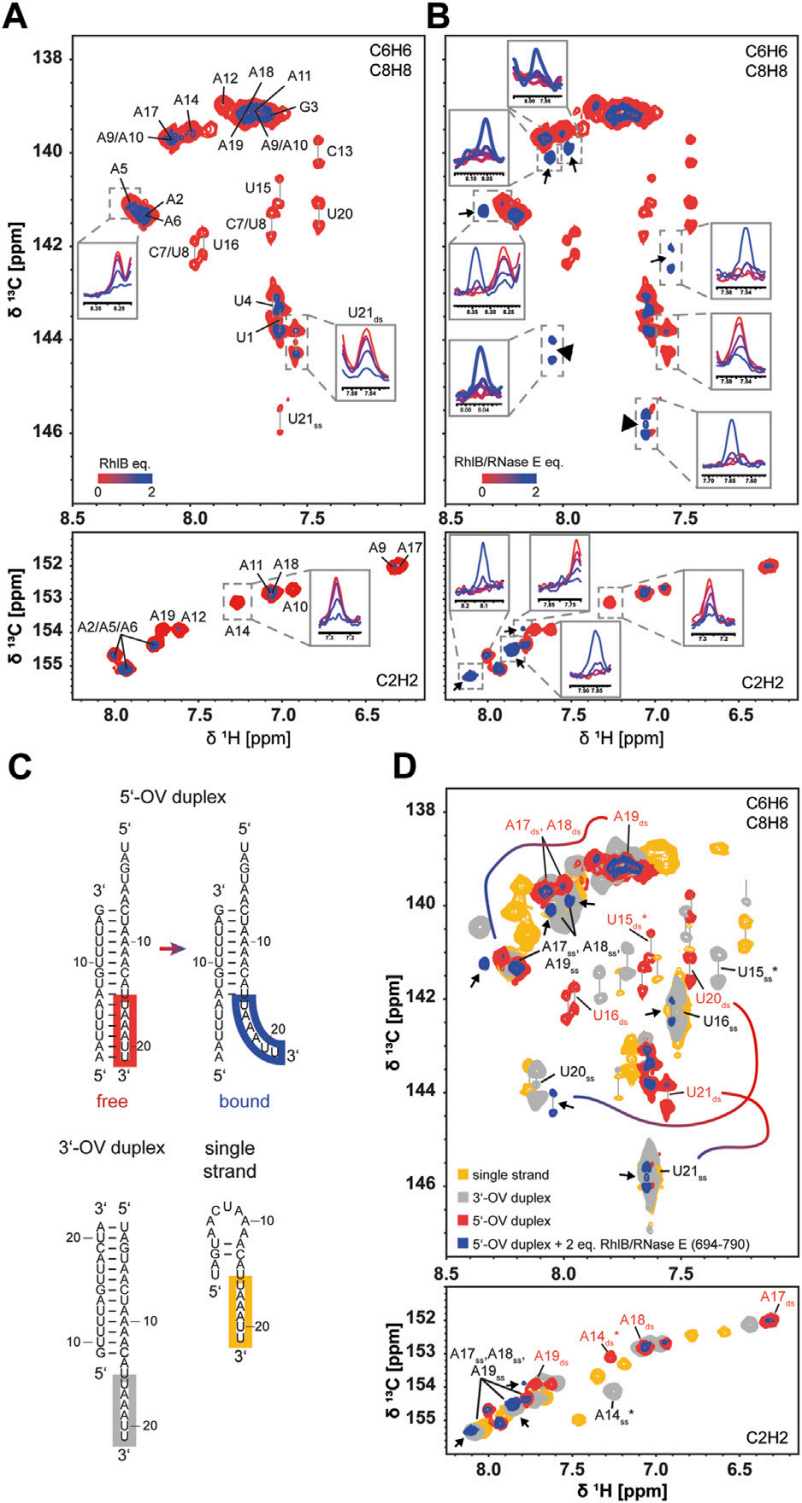
Conformation of RNA substrate during RhlB binding in absence and presence of RNase E (694-790). Superposition of ^13^C HSQC spectra of the 5’-OV RNA duplex titrated with up to 2 equivalents of RhlB **(A)** or RhlB/RNase E (694-790) **(B)**. Overlayed are the spectra of the aromatic regions for 0 (red) and 2 (blue) equivalents protein over RNA with exemplary 1D projections for each titration step to indicate the overall signal decrease. Spectra were recorded with 52 scans at 700 and 800 MHz for RhlB and RhlB/RNase E (694-790), respectively, at 288 K. Titrations were performed with 100 µM 5’-OV RNA substrate, that was exclusively ^13^C labelled in the 21 nt strand. **(B)** Newly arising signals are indicated with arrows and additional 1D projections for each titration step. **(C)** Structures of 3’-OV, 5’-OV and 21 nt single stranded RNA constructs as determined by NMR spectroscopy. Relevant nucleotides U21 to U16 are highlighted in the corresponding strands. **(D)** Superposition of ^13^C HSQC spectra from **(B)** with spectra of 100 µM 3’-OV duplex (grey) and 100 µM 21 nt single strand construct (yellow). ^13^C HSQC of the 3’-OV duplex was recorded at 288 K and 600 MHz with 32 scans, while the 21 nt single strand was recorded at 288 K and 700 MHz with 52 scans. Newly arising signals were again indicated with arrows and nucleotides corresponding to the base paired and unpaired conformation of U21 to U16 were labelled accordingly. U15 and A14 are marked with an asterisk (*), as they represent the limit in the RNA sequence were a spectral alignment of 3’-OV RNA and single strand was present due to structural similarity. For the full resonance assignment of both 5’-OV and 3’-OV duplex, see Supplementary Figure S4. For three exemplary resonance (U21, U20 and A19) the change in chemical shift stemming from the conformational change when bound to the helicase is indicated by the color gradient line. Similar changes are observed for all nucleotides boxed in panel **(C)**.

Stepwise addition of up to 2 equivalents of RhlB to the labelled 5’-OV RNA construct showed a homogenous intensity decrease throughout all monitored resonances comparable to the previously measured imino protons. In Figure 5A we overlayed the spectra of the first and last titration step and illustrated the stepwise decrease of the peak intensities with 1D projections of exemplary resonances. For a complete analysis of all resonances, see Supplementary Figure S3. No chemical shift perturbations could be observed for any nucleobase resonance, which indicated that there is no significant change in chemical surrounding or change in conformation experienced by the nucleobase resonances upon binding to the helicase.

Remarkably, we did observe significant differences in the titration with the RhlB/RNase E (694-790): while nucleobase resonances of the 5’-OV RNA duplex showed the same uniform intensity decrease as before, a new subset of peaks appeared, implying the formation of a conformationally distinct second population of nucleotide resonances upon binding (Figure 5B). Taken together, nine new peaks harbouring narrow linewidth and high intensity arise in the aromatic region of the spectrum. These new peaks are attributed to three pyrimidine (three new C6H6 peaks) and three adenine residues (three new C8H8 and C2H2 peaks). Due to their shape, they have to stem from a more flexible and possibly single stranded conformation. As the 3’-end of the longer strand reads 3’-UUAAAU, most probably these nucleotides are affected and reside as a single strand in the complex with RhlB/RNase E (694-790). To corroborate this assumption, we compared the 13C HSQC spectra with two more constructs that exhibit single strand extensions: the 3’-OV duplex and the 21 nt single strand. In both constructs, nucleotides A14 to U21 are unpaired and adopt conformations of single stranded RNA. Comparing the newly appearing resonances in the C6H6, C8H8 and C2H2 region of the titration endpoint (2 equivalents protein over RNA) with all three RNA constructs (Figure 5C) reveals a striking agreement in chemical shift with resonances of the unpaired 3’-tail. In case of the adenine C2H2 resonances, two of the three resonances directly overlap with signals that are assigned to unpaired adenines. The third upcoming adenine peak is slightly shifted but nonetheless resonates at a chemical shift typically reserved for adenines devoid of stable base pair formation. These peaks can be assigned to the three adenines in single stranded conformation A17ss, A18ss, and A19ss. In the C6H6/C8H8 region of the ^1^H,^13^C-HSQC spectrum three of the new peaks could be identified as pyrimidines due to their doublet splitting. Two of those match the resonances of unpaired U16ss and U21ss, while the third pyrimidine arises in direct proximity to the C6H6 of unpaired U20ss. The three remaining new resonances can be assigned as purine nucleotides and match the number of new C2H2 adenine signals. Here we can also see a significant overlap of two resonances with the unpaired adenines A17ss, A18ss and A19ss. A third peak is slightly downfield shifted compared to the third unpaired adenine resonance. Slight deviations in the chemical shift overlap between the new resonances and the 3’-tail of the 3’-OV duplex might arise from the RNA being bound to the protein, where interactions with the amino acids in the binding pocket affect the nucleotides locally.

It is known from various crystal structures of other DEAD-box helicases with single stranded RNA substrates that the amino acid coordination within the RNA binding pocket encompass 5 consecutive nucleotides (Sengoku et al., 2006; Linder and Jankowsky, 2011). This also indicates that the sequence of six unpaired nucleotides represent a single binding position within the binding pocket of RhlB. It should be noted that that under the given experimental conditions (buffer conditions and pH optimized for protein-RNA complex formation) the imino proton signals of U20, U21, U3, U4 are broadened beyond detectability, this is due to their inherent lower stability. However, the chemical shift range in the 13C-edited spectra and their line shape reports on the formation of base pairing interaction for all six terminal base pairs from A1-U21 to A6-U16 in the absence of RhlB/RNAse E.

In summary, the ^13^C-HSQC NMR spectra revealed a conformational transition in a stretch of six nucleotides of the 5’-OV duplex from a base paired to an unpaired conformation only upon binding to RhlB in complex with RNase E (694-790). The other part of the duplex however remains intact, as 1H NMR titration spectra did not show a complete loss of imino proton resonances even at 4 equivalents of protein for any RNA construct, as would be expected for strand separation (data not shown). Those findings provide a novel insight into the pathway of communication between RhlB and RNase E and the structural effects that are at the basis of the elevated ATPase activity in presence of RNase E. Moreover, our results provide the first evidence of a DEAD-Box helicase structurally changing the RNA substrate in absence of ATP.

## 4 Discussion

The presented substrate RNA centered study of RhlB’s helicase mechanism reveals that in absence of the interaction partner RNase E no substrate specificity is detectable for the ATPase activity. However, in the presence of different variants of RNase E an RNA topology dependent modulation of its activity can be determined. In line with data from Chandran et al., upon interaction with RNase E RhlB exhibits a clear preference for 5’-extended duplexes over blunt ended or 3’-extended substrate RNAs. The most unexpected findings are that the activity, as measured by ATP turnover, is largest for a single stranded RNA substrate and upon interaction of RNase E (628-843), a fragment that also contains RNA binding domains RBD and AR2 flanking the RhlB binding site. Earlier studies did not observe an increased ATPase activity through these parts of RNase E, as they worked with bulk RNA from *S. cerevisiae* comprising a mix of different topologies of RNA substrates (Khemici et al., 2004; Worrall et al., 2008), most probably resulting in an averaging out of their opposing effects on RhlB’s activity. Here, the increased activity might be induced by the single strand binding properties of both RBD and AR2, that scavenge the released single stranded RNAs at the end of the unwinding cycle. The single strand induced boost of the ATPase activity might also be rooted in a shift of the equilibrium towards the product site of the reaction, thereby enabling a low transition energy path through RhlB’s reaction cycle. It is important to note that although single stranded RNA is not to be unwound in the sense that base pairs must be broken, it is nevertheless a substrate for the helicase and induces conformational changes in the protein that are required for ATP hydrolysis. DEAD-Box helicases have not been studied using single stranded RNA substrates to assess their ATPase activity or RNA unwinding rate. Based on multiple published crystal structures of homologue DEAD-Box helicases utilizing ssRNA, it is known that single stranded substrates can be bound. As an example, one could speculate that an “unwinding reaction cycle” with a single stranded RNA would eliminate the step of dissociating the counter strand, thus increasing the reaction’s speed. There is also the possibility that RhlB prefers a specific tail-length on a duplex (similar results have been obtained for *E. coli* helicases SrmB, RhlE and CsdA (Bizebard et al., 2004)) and that based on the results of the ATPase assay our single-stranded RNA represents a substrate with a more favorable 5′end.

As revealed by our titration experiments, the activity of RhlB is actually matched with the affinities towards substrate RNAs with different strand topologies. These results are corroborated by the findings of Chandran et al. who reported similar substrate preferences in RNA unwinding assays (Chandran et al., 2007). Again, RNAs with 5’-OV exhibit the highest affinities towards RhlB compared to blunt ended duplexes or such with 3’-OV. The preference of the 5’-OV over the 3’-OV with respect to enzymatic activity and binding affinity is firstly a conclusive experimental finding. The molecular details that explain these findings are so far not experimentally validated. We assume that for the 5’-OV RNA the single stranded extension can better interact with RhlB via the C-terminal extension of the protein. However, this must be tested in future mutational studies.

For these cases, affinity is increased by ∼4 fold up to ∼10 fold upon formation of tertiary complex with RNase E (694-790) or (628–843), respectively. The increase in affinity cannot be explained by a simple additive effect, as at least the RNase E (694-790) fragment alone only exhibit a negligible affinity for RNAs. The modulation in affinity therefore must result from an RNase E induced conformational change within RhlB’s RNA binding site.

**FIGURE 6:**
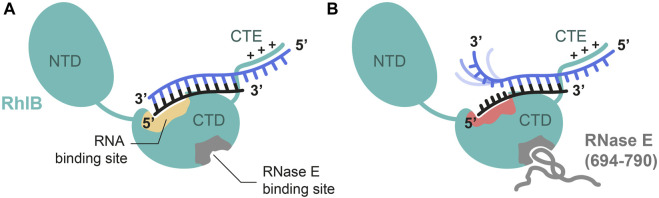
Proposed model of RhlB’s interaction with a 5’ tailed substrate RNA in absence **(A)** and presence **(B)** of RNase E (694-790). Schematic representation of RhlB in open conformation with bound RNA substrate in the absence of ATP. Binding sites for RNase E (grey) and RNA substrate (yellow) are highlighted within the C-terminal domain (CTD). The C-terminal extension (CTE) of RhlB interacts with the 5’ single strand extension of the RNA. Upon allosteric binding of RNase E (694-790) the interaction site is altered (red) in a way that the 5’ terminal nucleotides of the shorter strand (black) are bound. This leads to a separation of the terminal base pairs and leaves the 3’ end of the 21 nt strand (blue) dynamic and flexible.

Consequently, we investigated the formation of wild type RhlB in complex with RNase E (694-790) with a 15 nt RNA duplex featuring a 6 nt 5’-single strand tail in solution utilizing NMR spectroscopy. While previous studies predominantly gained information from crystal structures of DEAD-Box helicases in complex with poly-U RNA single strands, very little investigations have been performed to capture the complex in solution and with a physiologically more functional RNA substrate. In conjunction with the results from the activity and affinity measurements, the structural investigations lead us to propose the following model (Figure 6).

In the absence of protein interaction partner the RNA is bound to RhlB with reduced affinity and no opening of base pairing interactions can be observed. As RNAs with 5’-OV are preferentially bound compared to blunt ended double stranded RNAs or those featuring a 3’-OV, we propose that binding occurs at a binding site within C-terminal domain and further through an unspecific electrostatic but beneficial interaction between RhlB’s positively charged C-terminal extension (CTE) and the single stranded overhang. Although we cannot derive this interaction directly from our data, it is in line with earlier characterizations of multiple other DEAD-Box helicases also featuring a positively charged C-terminal extension (CTE) that demonstrate the assisting contribution of said extensions to RNA binding. Deletion of the CTE has also been shown to reduce RhlB’s RNA binding affinity, which strongly indicates its involvement in RNA binding (Chandran et al., 2007). The residence of the RNA binding site in the CTD is derived from findings, that in absence of substrates DEAD-box helicases adopt an open conformation, populating a conformational ensemble in which the two RecA-like domains are separate and have some independent mobility (Caruthers et al., 2000; Story et al., 2001; Theissen et al., 2008; Mallam et al., 2011). Binding studies of RhlB’s two individual domains have shown that the isolated C-terminal domain (CTD) is RNA binding competent, while the N-terminal domain itself does not show RNA binding capabilities (Chandran et al., 2007).

Upon interaction with RNase E (694-790), we infer an allosteric switch in the protein that ultimately leads to a conformational change in RhlB’s RNA binding pocket. The structural change in the binding pocket induces a partial opening of the blunt end of the 5’-OV RNA through the increased ability to interact with single stranded like conformations in the RNA. (Figure 6). With the RNase E (694-790) not being able to bind RNA individually, this structural change must be caused by the helicase itself in response to the allosteric binding of RNase E. The opening of base pairs in the substrate RNA is limited to a stretch of six nucleotides of the RNA substrate. Comparison with NMR spectra of the 3’-OV duplex and the 21 nt single strand revealed a striking alignment with the chemical shifts of the nucleotides U16 to U21 in an unpaired conformation. Since we still observed the broadened imino proton signals in the 1H NMR titration spectra even at a fourfold excess of protein, we can conclude that the remaining base pairs are still intact. To prevent those six separated base pairs from reannealing, at least one of the two RNA strands must be tightly coordinated by the helicase in the section of the blunt end in a way that is incompatible with duplex formation. While our selective isotope labelling scheme did not allow us to directly observe the nucleotide resonances of the shorter 15 nt strand, we can infer from the narrow linewidth and high signal intensity of the upcoming peaks that the unpaired 3’-end of the 21 nt strand is highly flexible and dynamic and thus not tightly bound. RhlB therefore has to interact with the nucleotides in the 5’-end of the 15 nt strand to maintain this partial duplex opening. This model is further supported by the results presented by Bruce and co-workers in 2018, where they showed in hydrogen-deuterium exchange analyses that binding of RNase E to RhlB causes reduced solvent exposure indicative of structural rearrangement for several areas exclusively in the CTD of the helicase. Those sites also encompassed the RNA binding motif IV and motif Va, which is responsible for communication between RNA and ATP binding (Bruce et al., 2018).

The allosteric activation of helicases is not unprecedented as, for example, a co-factor enhanced RNA unwinding could be shown for viral helicase NS3, that optimally performs its helicase activity on RNAs only upon binding to NS4a (Pang et al., 2002). In case of DEAD-Box helicases, only rare cases are reported, in which an allosteric modulator affects the helicases’ reaction activity by direct binding. Prominent examples are the mRNA exporting DEAD-Box helicase Dbp5, which can be conformationally locked in the inactive open state by the nucleoporin NUP214/Nup159, and eIF4A, which is locked in a half open conformation by eIF4G (Schütz et al., 2008; Von Moeller et al., 2009; Hilbert et al., 2011; Montpetit et al., 2011). However, here the allosteric change through RNase E binding to the ATP-free form of the helicase RhlB leads to a partial opened RNA conformation, ultimately increasing the helicases’ activity. Functionally this might reflect, that the interaction with the RNase lowers the activation barrier towards a RhlB complex with fully unwound RNA and therefore accelerates the next ATP-dependent conformational change occurring in the reaction cycle. Concluding we could show that the intricate network of interactions between RNase E, RhlB and substrate RNA directly influences the conformational state of the RNA. It will be interesting to see which additional protein-protein interactions drive or eventually inhibit the mode of function of this helicase central to the degradosome complex.

## Data Availability

The original contributions presented in the study are included in the article/Supplementary Material, further inquiries can be directed to the corresponding author.
