# Supplementary material for: Allosteric activation of RhlB by RNase E induces partial duplex opening in substrate RNA
Source: Front Mol Biosci. 2023 Aug 31;10:1139919. doi: 10.3389/fmolb.2023.1139919 (PMC10500059; doi:10.3389/fmolb.2023.1139919)
Supplement: Supplementary file 1 [file DataSheet1.PDF]

Allosteric activation of RhlB by RNase E induces partial duplex opening in substrate RNA

Heidi Zetzsche, Laura Raschke and Boris Fürtig*

Institute for Organic Chemistry and Chemical Biology, Center for Biomolecular Magnetic Resonance (BMRZ), Johann Wolfgang Goethe-Universität, Frankfurt am Main, Hessen 60438, Germany

* To whom correspondence should be addressed. Tel: +49 69 79829130; Email: fuertig@nmr.uni-frankfurt.de

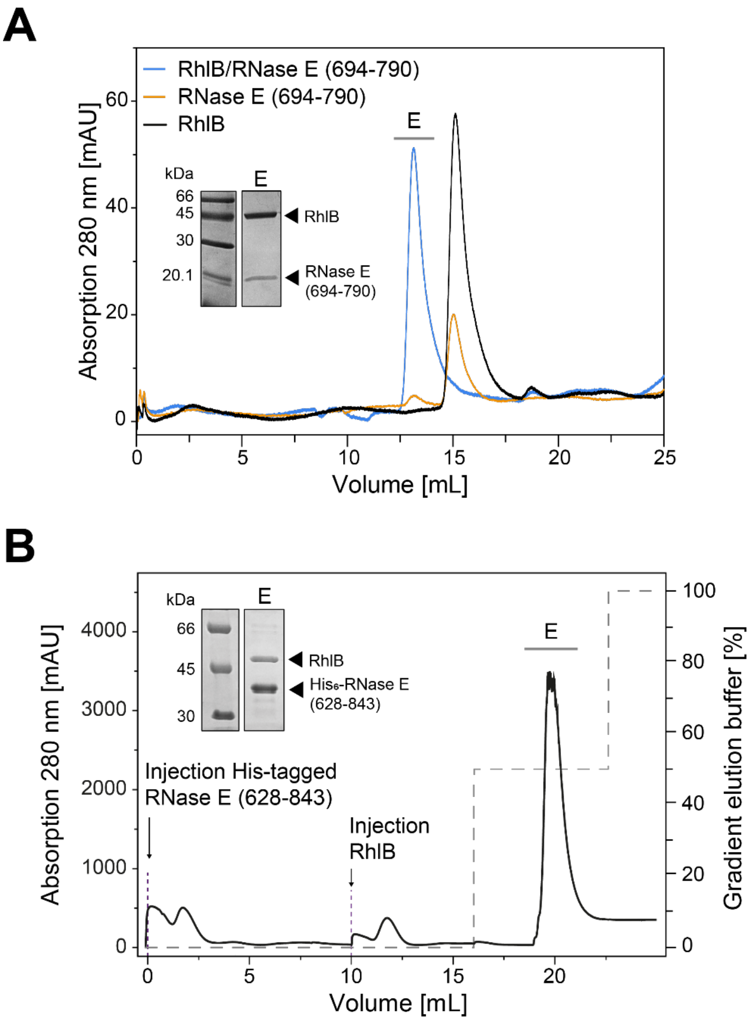

SI figure 1. Binding analysis of RhlB with RNase E (694-790) and RNase E (628-843). (A) S200 analytical size exclusion chromatograms of 100 µM RhlB, RNase E (694-790) and a 1:1 mix of both proteins. A clear shift in peak profile toward a higher molecular weight species could be observed for the mixed sample, which was identified as a complex of both proteins using SDS-PAGE. Note that the unstructured nature of RNase E (694-790) results in an abnormal running behaviour in both the size exclusion profile as well as the SDS PAGE: The RNase E fragment elutes approximately 3 mL earlier than a globular protein of the same size and migrates in the SDS-PAGE at the height of 20 kDa while being in fact 11 kDa in size. (B) Pulldown-assay using His_6-tagged RNase E (628-843) with untagged RhlB. Both proteins are sequentially loaded onto a 5 mL HisTrap Ni-affinity column and co-eluted using a gradient of elution buffer. The presence of both proteins in the elution fraction as verified via SDS-PAGE, demonstrates the binding of RhlB to its interaction partner.

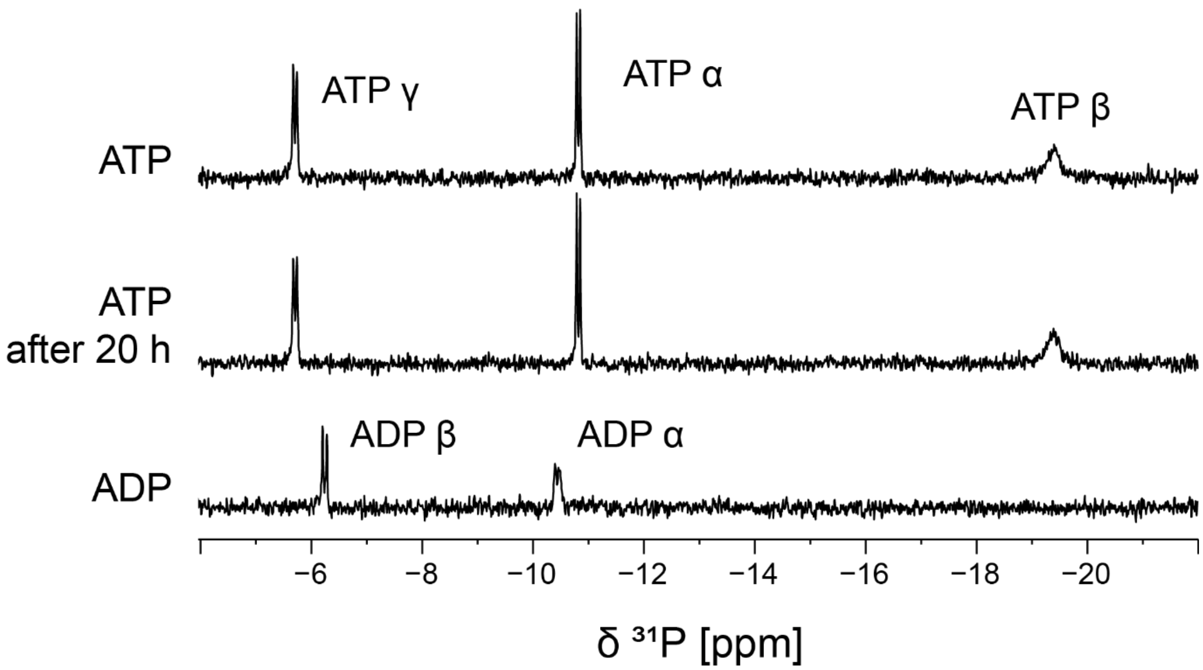

SI figure 2. ATP stability test. ^{31}P 1D spectra of 3 mM ATP in NMR buffer directly after preparation and 20 h after incubation at room temperature (295 K) as well as 100 µM ADP in NMR buffer for reference. The spectra clearly show that no spontaneous hydrolysis of ATP to ADP takes place under those buffer conditions, since no ADP signals could be detected in the ATP spectrum after 20 h. Spectra were recorded at 288 K and 600 MHz with 265 scans and a spectra width of 50 ppm.

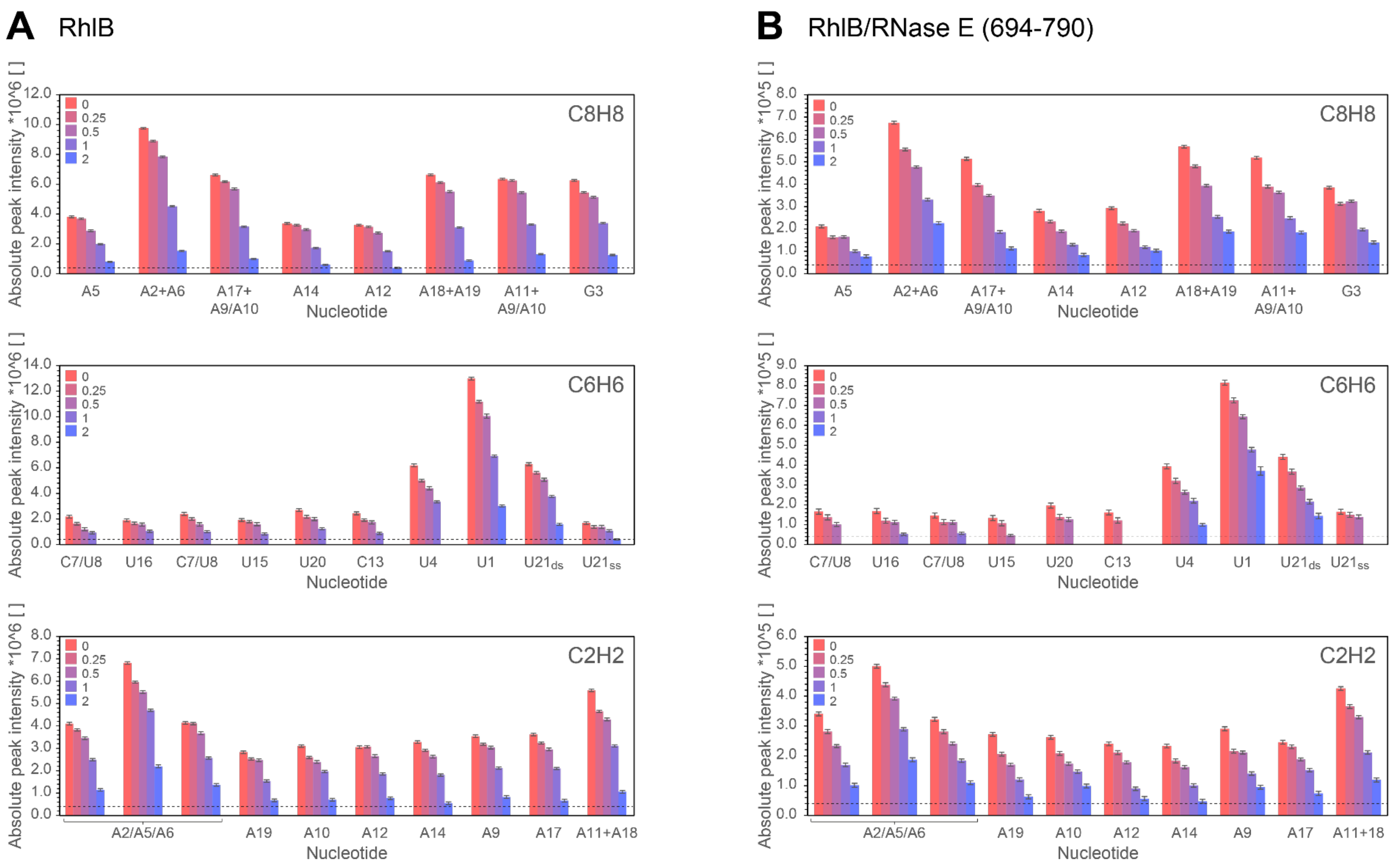
A RhlB
B RhlB/RNase E (694-790)
C8H8
C6H6
C2H2
Absolute peak intensity *10^6 [ ]
Absolute peak intensity *10^5 [ ]
Nucleotide
0
0.25
0.5
1
2
A5
A2+A6
A17+ A9/A10
A14
A12
A18+A19
A11+ A9/A10
G3
C7/U8
U16
U15
U20
C13
U4
U1
U21ds
U21ss
A2/A5/A6
A19
A10
A9
A17
A11+A18
A11+18

SI figure 3. Nucleobase peak intensities of 100 µM 5'-OV RNA during titration with up to 2 equivalents of RhlB or RhlB/RNase E (694-790). ^{13}C HSQC spectra of each titration step recorded with 52 scans at 700 and 800 MHz for RhlB and RhlB/RNase E (694-790), respectively, at 288 K. Spectra were referenced against the chemical shift of DSS. Peak intensities were extracted from Sparky and errors calculated from the S/N ratio of the corresponding spectra. The dashed lines represent the sensitivity threshold, at which the peaks were detected. Resonance assignment is matched with the assignment depicted in SI figure 4.

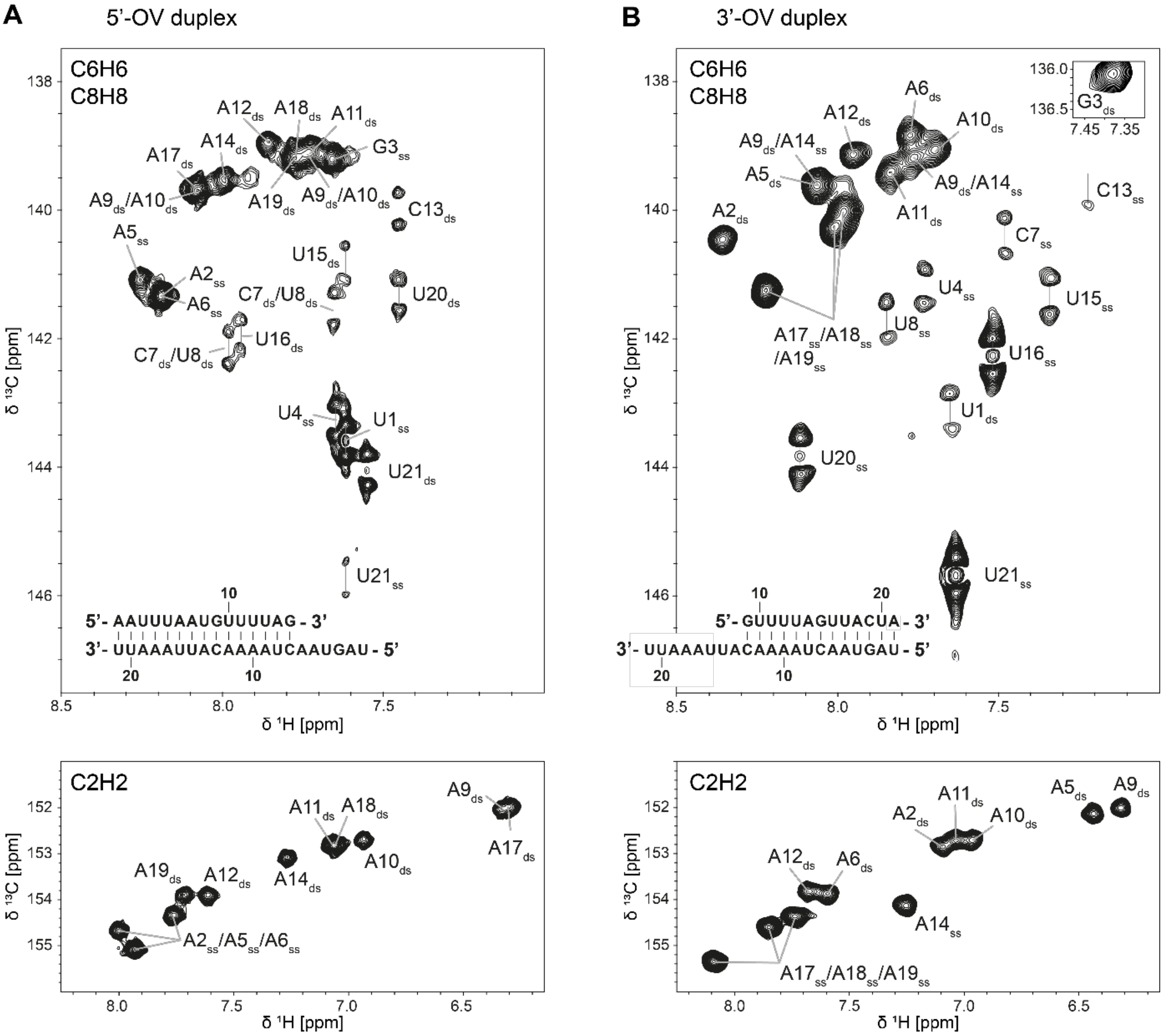

SI figure 4. Full assignment for C2H2, C6H6 and C8H8 nucleobase resonances of 5'-OV duplex (A) and 3'-OV duplex RNA (B). In both constructs the 21 nt bottom strand was fully ^{13}C labelled, while the shorter top strands were unlabelled. The depicted assignments therefore correspond to nucleotides in the 21 nt strand. Where an unambiguous assignment was not possible due to spectral overlap, the labels depict all possible assignments separated by slashes. The annotations ds and ss correspond to the double stranded and single stranded conformation, respectively.

3’OV duplex + RhlB

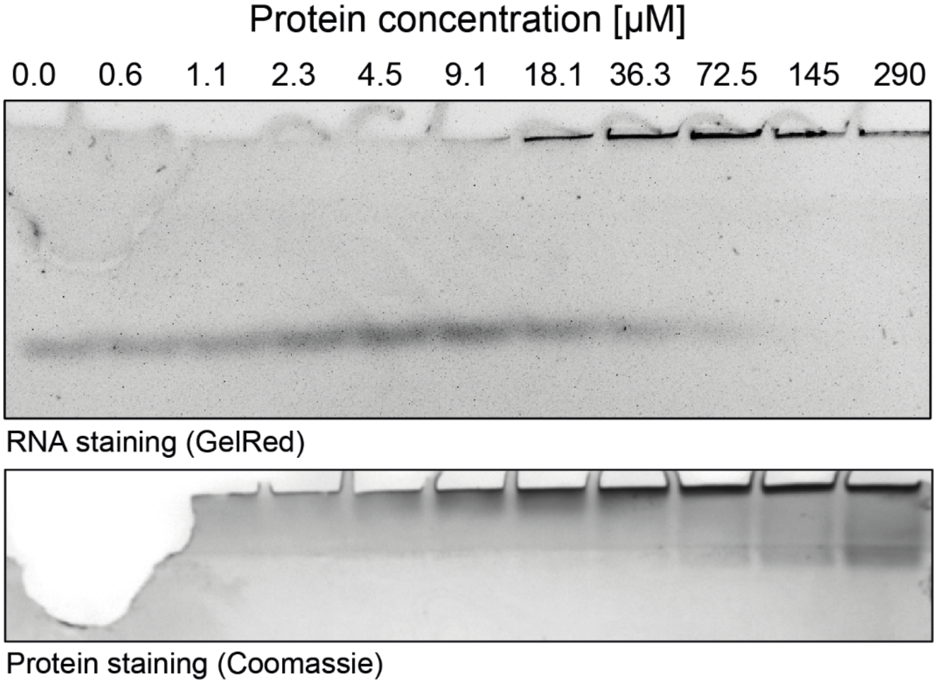

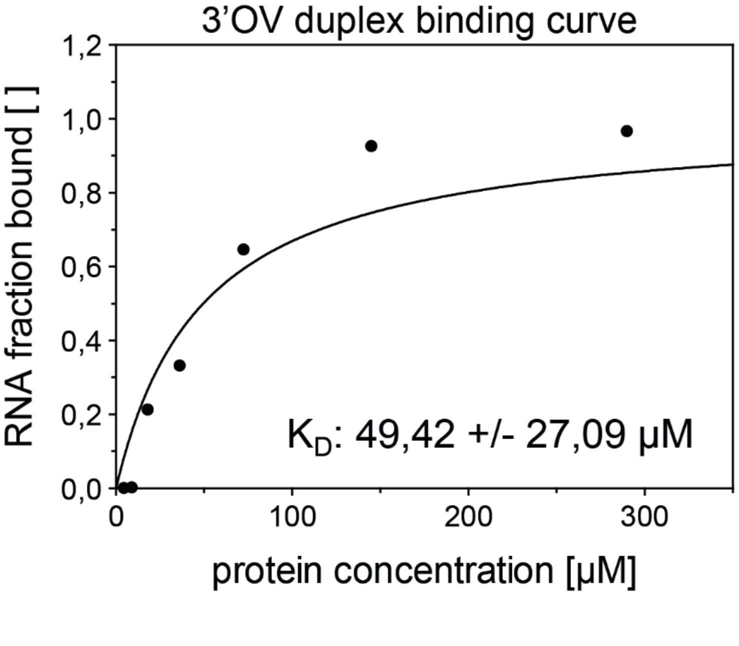

SI Figure 5. Electrophoretic mobility shift assays with 3’OV RNA duplex alone and with increasing concentrations of RhlB. The protein concentration is increased from 0.6 to 290 µM as indicated for the individual gel lanes. The free RNA is visible as a single band on the gel, while the protein-RNA complex is remaining in the gel pocket as the helicase does not migrate into the gel under the given native PAGE conditions. The gels were stained with GelRed for RNA visualisation (upper gel panel) and Coomassie for protein visualisation (lower gel panel). Quantification of the free RNA with ImageJ was performed as described in Materials and Methods. The fraction of RNA in complex determined in gel shift assays plotted as a function of protein concentration. Curves are best fit to a simple single binding site model. Due to slightly irregular gel staining some datapoints were omitted from the analysis to obtain a more accurate curve fit.
